# Construction of a Microsatellites-Based Linkage Map for the White Grouper (*Epinephelus aeneus*)

**DOI:** 10.1534/g3.114.011387

**Published:** 2014-06-05

**Authors:** Lior Dor, Andrey Shirak, Sergei Gorshkov, Mark R. Band, Abraham Korol, Yefim Ronin, Arie Curzon, Gideon Hulata, Eyal Seroussi, Micha Ron

**Affiliations:** * Institute of Animal Science, Agricultural Research Organization, Bet Dagan 50250, Israel; † Robert H. Smith Faculty of Agriculture, Food and Environment, Hebrew University of Jerusalem, Rehovot 76100, Israel; ‡ National Center for Mariculture, Israel Oceanographic and Limnological Research, Eilat 88112, Israel; § The Carver Biotechnology Center, University of Illinois, Urbana, Illinois 61801; ** University Haifa, Institute of Evolution, of Haifa 3498838, Israel

**Keywords:** *Epinephelus aeneus*, Linkage map, Microsatellite markers, Comparative mapping

## Abstract

The white grouper (*Epinephelus aeneus*) is a promising candidate for domestication and aquaculture due to its fast growth, excellent taste, and high market price. A linkage map is an essential framework for mapping quantitative trait loci for economic traits and the study of genome evolution. DNA of a single individual was deep-sequenced, and microsatellite markers were identified in 177 of the largest scaffolds of the sequence assembly. The success rate of developing polymorphic homologous markers was 94.9% compared with 63.1% of heterologous markers from other grouper species. Of the 12 adult mature fish present in the broodstock tank, two males and two females were identified as parents of the assigned offspring by parenthood analysis using 34 heterologous markers. A single full-sib family of 48 individuals was established for the construction of first-generation linkage maps based on genotyping data of 222 microsatellites. The markers were assigned to 24 linkage groups in accordance to the 24 chromosomal pairs. The female and male maps consisting of 203 and 202 markers spanned 1053 and 886 cM, with an average intermarker distance of 5.8 and 5.0 cM, respectively. Mapping of markers to linkage groups ends was enriched by using markers originating from scaffolds harboring telomeric repeat-containing RNA. Comparative mapping showed high synteny relationships among the white grouper, kelp grouper (*E. bruneus*), orange-spotted grouper *(E. coioides*), and Nile tilapia *(Oreochromis niloticus*). Thus, it would be useful to integrate the markers that were developed for different groupers, depending on sharing of sequence data, into a comprehensive consensus map.

The white grouper (*Epinephelus aeneus*) is a marine teleost fish distributed along the Atlantic west coast of Africa and the southern Mediterranean Sea (Heemstra and Randall 1993). The genus is one of 15 genera of subfamily *Epinephelinae*, including 159 species commonly defined as groupers (Maggio *et al.* 2005). Initially, groupers were identified and taxonomically classified based on morphologic characteristics (Heemstra and Randall 1993). Recent studies explored the taxonomic status and evolutionary relationships among several species of groupers using molecular markers such as random amplified polymorphic DNA (Govindaraju and Jayasankar 2004), and mitochondrial cytochrome b, mitochondrial 16S rDNA, and cytochrome oxidase subunit I (Maggio *et al.* 2005; Dor *et al.* 2014).

Like many other groupers, the white grouper is a promising candidate for intensive aquaculture due to its high market value, excellent taste, and rapid growth rate (Hassin *et al.* 1997; Glamuzina *et al.* 2000). The first attempt to domesticate the white grouper in Israel was carried out during the late 1990s. The research focused mainly on growth rate and development of induced reproduction techniques (Hassin *et al.* 1997). At present, farming of the white grouper is an emerging sector of the Israeli aquaculture. The main captive breeding stocks are based on wild populations captured from the natural environment, and no domesticated strains resulting from breeding are yet available. A white grouper domestication project was launched by the Department of Fishery and Aquaculture of the Israeli Ministry of Agriculture in 2010. The mission of the project is to develop innovative technologies aimed at domestication, breeding, and industrial rearing of the white grouper (Gorshkov 2010; Meiri-Ashkenazi *et al.* 2010).

Despite of intensive studies in breeding and larviculture techniques of the white grouper, low and unstable survival of larvae before metamorphoses is still a major problem (Gorshkova *et al.* 2002; Gorshkov 2010). Similar problems have also been indicated at the early larval stages in the cultured red-spotted grouper (*Epinephelus akaara*) (Yamaoka *et al.* 2000; Okumura *et al.* 2002).

The reproductive behavior of the white grouper is not fully understood. Unlike some other groupers, *e.g.*, the dusky grouper (Zabala *et al.* 1997) and the halfmoon grouper (Mackie 2007), white grouper cannot be propagated in pairs, and the common practice is to spawn them in groups inside breeding tanks. It has recently been discovered that only a few matured individuals in a broodstock actively contribute genetic material to their offspring (Dor *et al.* 2014). This can dramatically reduce the genetic diversity over generations. Thus, appropriate breeding schemes should be adopted for genetic management of captive broodstock of the white grouper to minimize inbreeding.

Microsatellite markers are used widely for parenthood analyses, population genetics studies, and selection of agricultural stocks due to their genome abundance and high level of polymorphism (Tautz *et al.* 1986; Jones *et al.* 2010). In a previous study we examined heterologous microsatellite markers originating from different grouper species and found that 42–83%, depending on phylogenetic distances among species, are conserved and polymorphic in the white grouper (Dor *et al.* 2014).

A genetic linkage map is an important resource for marker-assisted selection, domestication, and management of aquaculture important species (Liu and Cordes 2004). Many genetic maps have been constructed for Perciformes species such as the Nile tilapia *(Oreochromis niloticus*) (Lee *et al.* 2005), European sea bass *(Dicentrarchus labrax*) (Chistiakov *et al.* 2005), Asian seabass *(Lates calcarifer*) (Wang *et al.* 2011), the guilthead sea bream *(Sparus aurata*) (Franch *et al.* 2006), kelp (longtooth) grouper *(Epinephelus bruneus*) (Liu *et al.* 2013b), and orange-spotted grouper (*Epinephelus coioides*) (You *et al.* 2013). Using linkage maps, candidate genes and quantitative trait loci (QTL) affecting economically important traits can be detected by association studies (Cnaani *et al.* 2003; Shirak *et al.* 2006). Deep-sequencing technology may facilitate the design of microsatellite markers (Wang *et al.* 2012). Assembly of the genome into scaffolds can significantly improve the efficiency of linkage map construction by allowing the design of uniformly spaced markers. Like other vertebrates, fish chromosome telomeres consist of telomeric DNA that are capable of encoding repeat-containing RNA (TERRA), which are tandem repeats of the TTAGGG sequence motif (Lejnine *et al.* 1995). Scaffolds containing TERRA tandem repeats can be used to enrich mapping of markers to the chromosomal telomeres.

We report here the identification of genetic markers in the white grouper using deep sequencing data and the construction of the first-generation linkage map for this species. This map was compared for synteny with the maps of kelp (longtooth) and orange-spotted groupers and tilapia.

## Materials and Methods

### Sampling and DNA extraction

Forty-eight and 32 three-month-old grouper progeny were sampled from two subsequent spawns in a breeding tank containing broodstock of 12 fish. DNA of parental broodstock and their progeny was extracted from blood or whole-body tissue using MasterPure DNA Purification Kit (Epicentre Biotechnologies, Madison, WI) following the manufacturer’s recommended protocol.

### Deep sequencing and assembly of the white grouper genome

DNA of a single offspring from the mapping population was sequenced using Illumina 100-bp paired-end technology (HiSequation 2000; Illumina, San Diego, CA). The sequencing quality was uniformly high, with sample mean PHRED score in a narrow band around 34–41. The genome reads were assembled using SOAPdenovo2 modules (http://sourceforge.net/projects/soapdenovo2) in four steps as follows: (1) the data were trimmed with TrimmomaticPE (http://manned.org/TrimmomaticPE) using the following parameters: LEADING:30, TRAILING:30, MINLEN:50; (2) the frequency of Kmers in the trimmed output was calculated using the SOAPdenovo2 module KmerFreq_AR under the options -k 17 -t 7 -q 33; (3) the output of the KmerFreq_AR module was used to correct sequence errors in the data reads using the Corrector_AR module with -r 30 -t 7 switches; and (4) the sequence reads were finally assembled applying the command line “SOAPdenovo-63mer all -s config_file -K 35 -m 51 -p 8.” The config_file included the lines: max_rd_len = 100, avg_ins = 600, reverse_seq = 0, asm_flags = 3, rank = 1 and the access paths for the files with the trimmed and the corrected sequence reads of the forward and reverse mate pairs (q1, q2). Output scaffolds were sorted according to their sequence length and numbered accordingly from largest to smallest.

### Construction of a mapping family

DNA of 80 progeny was quantified and diluted to uniform concentration of 10 ng/µl. DNA samples were distributed into 96-well polymerase chain reaction (PCR) plates and dried. Amplification of microsatellites was performed by a two-step PCR reaction according to Dor *et al.* (2014) with minor modification: uniform annealing temperature of 55° was elevated to 57° and 60° for the first and second PCR steps, respectively. All 80 samples of offspring were genotyped for 34 microsatellites that were previously found polymorphic in the captive broodstock and tested on its previous season’s spawns populations (Dor *et al.* 2014). These 34 markers were selected based on their high polymorphism rate of at least three alleles in the 12 potential parents. All 80 progeny were assigned to parents according to segregation of markers. A total of 48 offspring of a single parental pair were selected to constitute the mapping family, for construction of a first-generation linkage map based on genotyping data of 228 microsatellites.

### Design of microsatellite markers

Novel microsatellite markers were designed in the 177 largest scaffolds based on DNA sequence of the assembled grouper genome. In addition, using local BLAST (blastall 2.2.25, Camacho *et al.* 2009), 11 scaffolds containing the repetitive sequence (TTAGGG)_5_ were selected for enrichment of mapping markers to the telomeres (Lejnine *et al.* 1995). Microsatellites were constructed by a 4-step procedure: (1) identification of loci that contained (GT)_10_ repeat motif; (2) selection of those loci that contained ambiguous sequences (NNNNN) adjacent to the GT repeats; (3) selection of loci that do not contain additional repetitive elements (GIRI, http://www.girinst.org/censor/index.php and Tilapia Repeat Masker, http://cowry.agri.huji.ac.il/cgi-bin/TilapiaRM.cgi); and (4) design of PCR primers within 200 bp regions that flank the GT repeats (PRIMER3, http://bioinfo.ut.ee/primer3-0.4.0/).

### Nomenclature of microsatellite markers

The scaffolds were assigned numbers from largest to smallest. Microsatellites were named with the prefix “D” followed by the number of scaffold on which they reside, *e.g.*, from D001 to D200. D002, D009, D046, and microsatellites with numbers >200 were constructed from scaffolds containing TERRA telomeric sequences. Microsatellites with the prefix “ARO” are heterologous markers (Dor *et al.* 2014) of which nine reside on large scaffolds (D001 through D200) (Supporting Information, Table S1).

### Genotyping of microsatellites

A total of 228 microsatellites were selected for genotyping, including a set of 40 heterologous markers (Table S1). The microsatellites were tested for polymorphism in parents of the mapping family (designated as M2 and F9) and genotyped for the 48 offspring by fragment analysis (GeneMapper software v.4.0, Applied Biosystems, Carlsbad, CA). Sets of bins were designed for each marker based on the parental alleles, and automatic allele calling of microsatellites for progeny was enabled (Figure S1).

### Analysis of synteny between white grouper and Nile tilapia

Sequences of the 177 mapped largest scaffolds of the white grouper were BLASTN searched against Nile tilapia (*Oreochromis niloticus*) sequences in the Broad anchored database (v1.1) (http://cichlid.umd.edu/blast/blast.html).

### Analysis of synteny between white, kelp, and orange-spotted groupers

Two hundred twenty-two sequences (350−500 bases) that were used for development of microsatellite markers in kelp grouper (Liu *et al.* 2013b), and 5455 sequences (84 bases) of single-nucleotide polymorphism (SNP) markers in orange-spotted grouper (You *et al.* 2013), were masked using GIRI and Tilapia Repeat Masker (Shirak *et al.* 2010) prior to BLASTN search against our white grouper assembled genomic database. These sequences were downloaded from the National Center for Biotechnology Information nucleotide database and from the supporting information of the aforementioned publications.

### Construction of a white grouper linkage maps

Genotyping results of 48 full-sibs and their parents for 228 microsatellite markers were analyzed by MultiPoint software (http://www.multiqtl.com/). The performed mapping analysis should take into consideration sex differences in recombination, a known phenomenon across different taxa, including fish species. Simultaneous estimation of female and male recombination rates from intercross data in outbred organisms is an easy task if the parental gametes can be deduced from the progeny genotypes, *i.e.*, when at each of the two marker loci 3−4 alleles are observed in the progeny. Maximum likelihood analysis enables the estimation of sex-specific recombination rates even if the parental gametes cannot be directly recovered from genotypes of the progeny, like in intervals with 2 rather than 3−4 alleles for one of the two linked loci (such markers can be referred to as F2 markers) (Lawrence *et al.* 1979). However, this may not be sufficient for building genetic maps, when two or more F2 markers are adjacent on the same linkage group. A specific algorithm implemented in MultiPoint software enables the assignment of male and female recombination rates between such F2 markers based on joint analysis of these markers with neighbor 3- or 4-allele markers and/or backcross markers (when only one of the parent is the source of segregating alleles). Based on the calculated matrices of rm and rf values for all pairs of markers, we constructed the corresponding female and male maps using the algorithm described in Mester *et al.* (2003), with some modifications caused by the special features of intercross data. In addition to the estimation of recombination between two markers of the F2 type, the analysis should fit the request of the same order of shared markers in the male and female maps. This is achieved by building a special variant of consensus mapping (Mester *et al.* 2010) implemented in MultiPoint (www.multiqtl.com). The constructed linkage groups (LGs) were drawn and aligned using MapChart v2.2 (http://www.wageningenur.nl/en/show/Mapchart.htm).

### Statistical analysis

The χ^2^ test was used to identify markers with a significant deviation from the expected Mendelian genotype segregation (http://vassarstats.net/csfit.html). This categorical test was also applied for enrichment analysis of mapping TERRA containing markers to linkage groups ends, and for comparison between success rate of hetero- and homologous polymorphic markers.

## Results

### Construction of a mapping family

Thirty-seven and 15 individuals in two subsequent spawns, respectively, were verified by segregation of genetic markers as offspring of a single pair of parents (M2 and F9). Furthermore, eight polymorphic markers (ARO *1045*, *1078*, *1087*, *1120*, *1124*, *1130*, *1137*, and *1145*) were sufficient for exclusion of the remaining 28 individuals with conflicting genotypes for at least two markers (Figure S2). These individuals were classified as offspring of an additional male (M4) and two females (F9 and F11) (Table S2). M2 was the dominant sire in both spawns with a total of 72 progeny (90%). F9 was the dominant dam in the first spawn (87% of progeny) but not in the second spawn (47% of progeny) (Table S2). Thus, 48 progeny of the dominant pair of parents (M2xF9) were selected to constitute the mapping family.

### Sequencing of the white grouper genome

Genomic sequencing of a single lane on the HiSeq2000 produced more than 387.5 million paired reads with sequence fragment average of 580 bases. SOAPdenovo2 assembled 94.5% of these reads into 556,710 scaffolds with mean scaffold size of 2219 bases. The total size of the white grouper genome was estimated at 1.1−1.2 Gb, with 32−34X coverage in this study. The longest scaffold had 913,988 bases, and 2294 additional scaffolds had more than 100,000 bases each. The total length of the 177 largest scaffolds, consisting of 269 to 914 Kb, was 72 Mb, representing approximately 6.5% of the white grouper genome.

### Marker design and polymorphism

A search for (GT)_10_ sequence motif in the largest 177 scaffolds detected 2248 matches with frequency of one occurrence per 28 Kb. In 1973 cases (88%) (GT)_10_ core repeats were adjacent to an ambiguous sequence denoted by large number of N letters that may indicate putative polymorphism. No additional repetitive elements were detected in 670 of them. Thus, these sequences were selected for PCR primer design. A single marker was designed for each of the 177 scaffolds. BLAST-search of the (TTAGGG)_5_ sequence (TERRA) against the genome assembly detected 42 positive sequences, but most of them were short contigs. Eleven of these sequences contained (GT)_10_ repeat motif and were selected for PCR primer design. By adding the 40 heterologous markers reported earlier, a total of 228 markers were selected for genotyping the mapping family (Table S3).

Polymorphism level of 2.4 alleles per marker was obtained. An example of a Hardy-Weinberg segregation of alleles for a single marker from parents to progeny is displayed in Figure S1. With a maximum of four possible alleles in parents, five types of informative allele segregation are expected in progeny of the mapping family (Table 1). Both parents were heterozygous for 190 of the 228 markers (83.3%), thus providing information for construction of both male and female linkage maps.

**Table 1:** Polymorphism of markers and type of segregation in the mapping family

No. Alleles	Type of Segregation*^a^*	No. Markers	Frequency, %
2	ABxAB	106	46.5
2	AAxAB	35	15.4
3	ABxAC	74	32.5
3	AAxBC	3	1.3
4	ABxCD	10	4.4
Total		228	

a Different alleles per marker are denoted by different letters (A−D).

### Construction of the white grouper linkage maps

**Figure 1:**
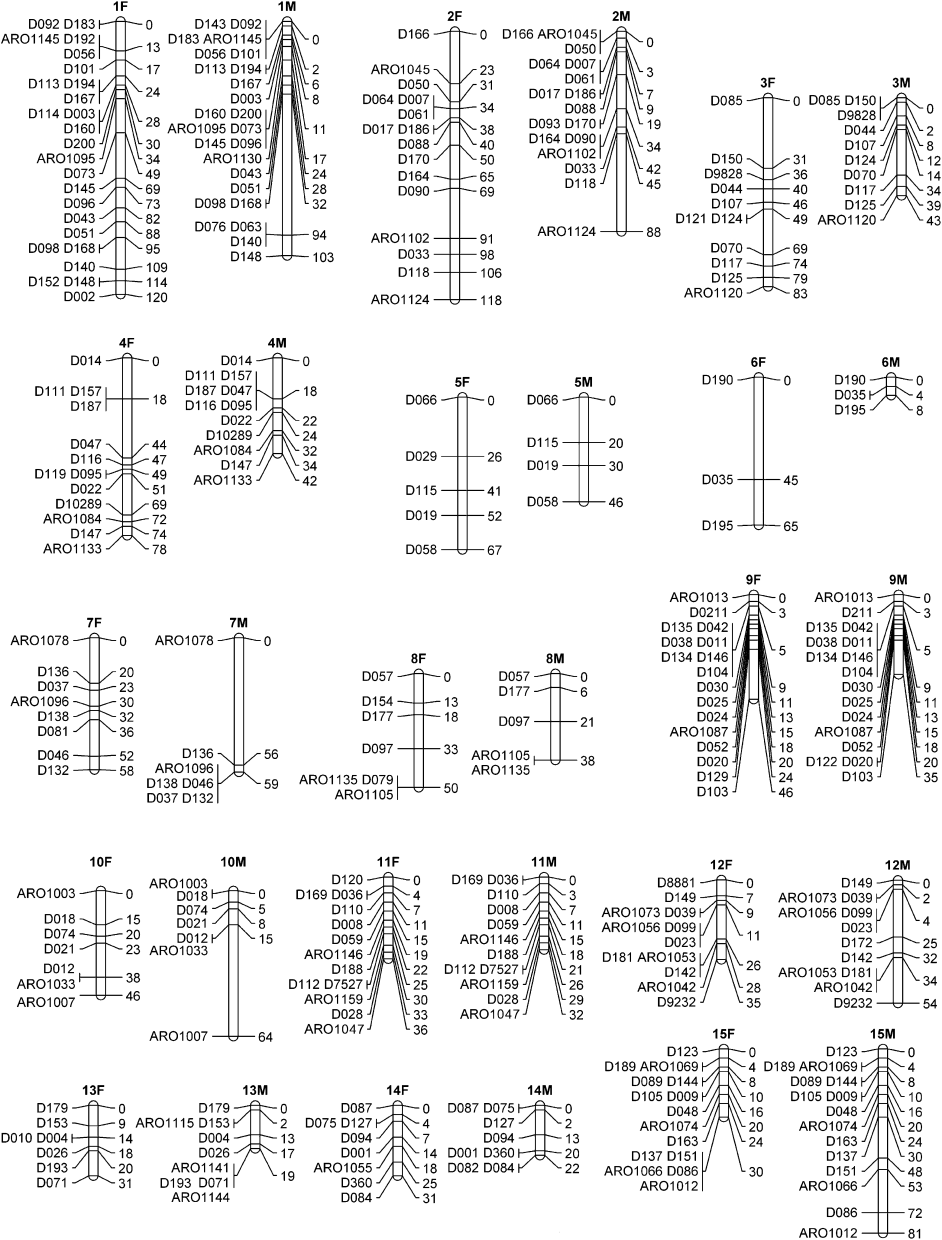
Sex-specific linkage maps of white grouper (LGs 1−15). The female (F) and male (M) linkage groups are presented side by side. Marker names and positions (Kosambi centiMorgans) are indicated on the left and right sides of the linkage group delineations, respectively.

**Figure 2:**
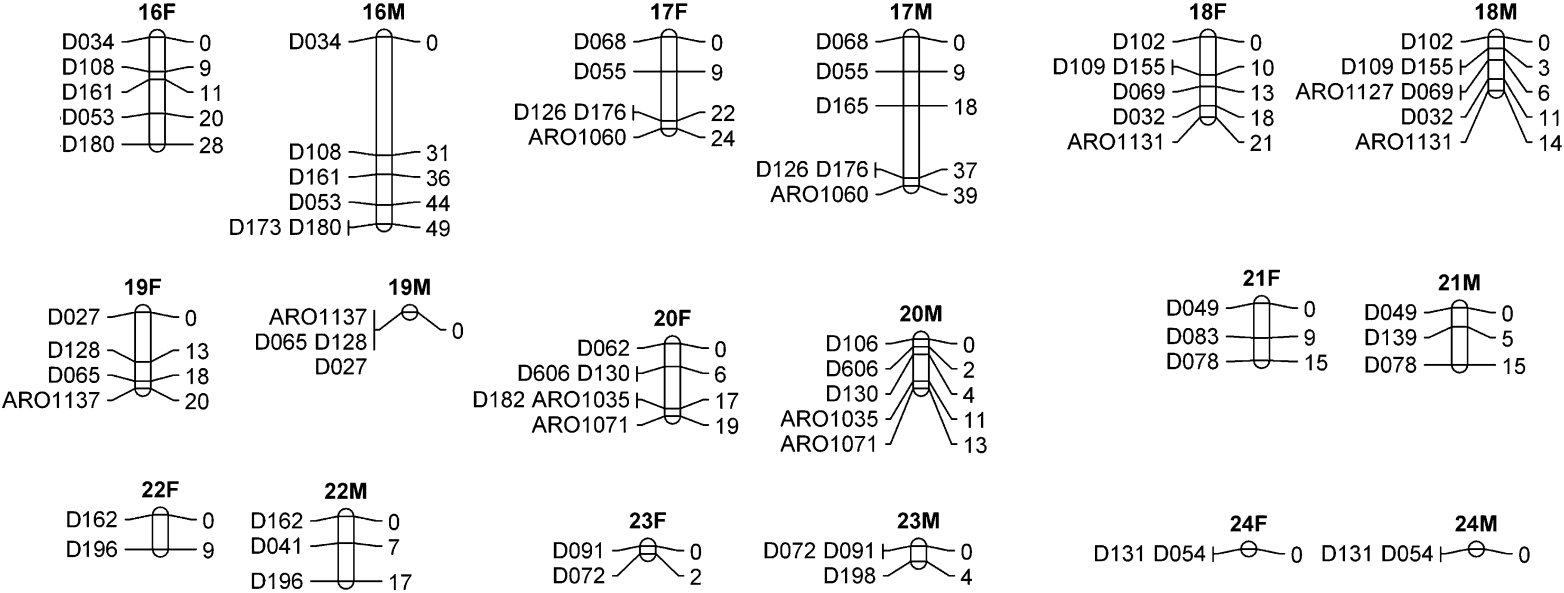
Sex-specific linkage maps of white grouper (LGs 16−24). The female (F) and male (M) linkage groups are presented side by side. Marker names and positions (Kosambi centiMorgans) are indicated on the left and right sides of the linkage group delineations, respectively.

**Figure 3:**
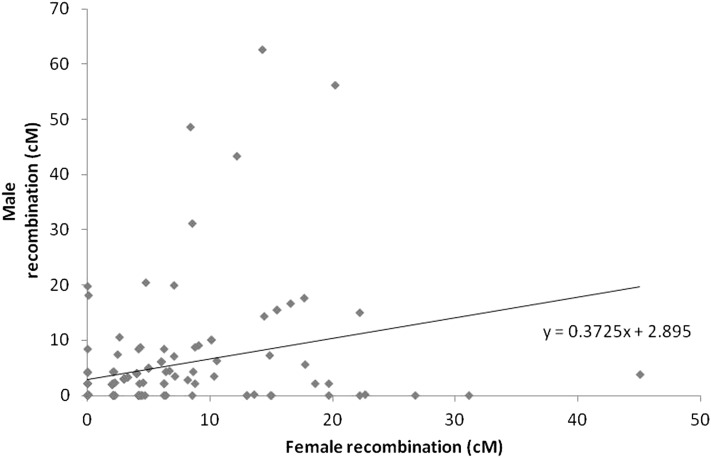
Intervals between adjacent markers (cM) in the male map were plotted against the respective size of intervals in the female map. The linear regression of male on female intervals was significant at *P* = 0.0008.

The MultiPoint software integrated 223 of the 228 microsatellite markers into female and/or male linkage maps depending on the segregating alleles in male and female parents. Thus, 203 and 202 markers were informative for the construction of female and male linkage maps, respectively, resulting in 24 linkage groups. The total lengths of the maps were 1053 and 886 cM for female and male, respectively. Thus, the average ratio of recombination between female and male was 1.19:1. Linkage groups were numbered in accordance to their length on the female map from the longest spanning 120 cM and containing 25 markers (LG1) to the shortest of virtually 0 cM containing two markers (LG24). The sex-specific LG maps are presented in Figure 1 and Figure 2. Intervals between adjacent markers were calculated in centimorgan units for both sex-specific maps using Kosambi mapping function. A total of 147 of the 178 intervals were for the same adjacent markers in both male and female maps, indicating that the same order of markers in linkage groups is maintained in both maps for most of the markers (83%). The distances in male and female maps are presented in Figure 3 for the common 147 intervals. It is evident that the male map is compressed in comparison with the female map, with a significant regression slope of 0.37 (*P* = 0.0008).

### Segregation distortion

Five of the 228 markers exhibited significant deviation from the expected Mendelian segregation (*P* < 0.01). These markers were mapped to five different LGs (Table 2). Analysis of segregation of neighboring markers supported the existence of segregation distortion in LGs 1 and 4. The first locus with segregation distortion was mapped to the center of the largest linkage group (LG1) with its maximum impact represented by marker D073. The genotypes for D073 were heterozygous for the same alleles in both of the parents, and segregated in their progeny at a ratio of 28:11:9 for AA:AB:BB, respectively. A decay of segregation distortion was observed in the two neighboring markers, *e.g.*, ARO1095 and D200, showing skewed segregation (*P* = 0.02). The second locus with segregation distortion was mapped to the center of LG4 with its maximum impact represented by marker D10282. A decay of segregation distortion was observed in the three neighboring markers, D116, D045 and D022, showing skewed segregation (*P* = 0.02−0.03). Segregation distortion of markers D085, D066, and D010 on LGs 3, 5, and 13, respectively, was not supported by cosegregation of adjacent markers.

**Table 2:** Non-Mendelian segregation of markers

Marker	LG	Type of Segregation	Deleterious Genotype	Distortion Probability*^a^*
D073	1	ABxAB	BB	<0.0001
D085	3	ABxAB	BB	<0.0001
D10282	4	ABxAC	BC	0.0096
D066	5	ABxAB	BB	<0.0001
D010	13	AAxAB	AB	0.0001

a χ^2^ test.

### Macrosynteny between the white grouper and Nile tilapia

**Figure 4:**
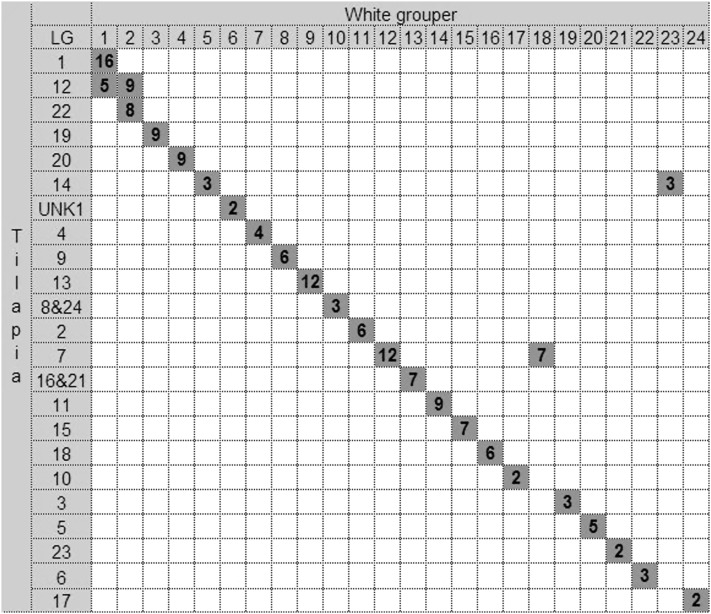
Macrosynteny relationships between linkage groups of white grouper and tilapia. The numbers of grouper markers with significant BLASTN hits in tilapia are presented in the table, and the putative syntenic pairs are indicated by gray boxes along the diagonal. The off diagonal gray boxes represent synteny to additional LGs. UNK1 represents a major fragment in the tilapia genome of unknown location. Both LGs 8 and 24 and LGs 16 and 21 were united according to the tilapia second linkage map (Guyon *et al.* 2012).

BLASTN search of the 177 largest scaffolds of white grouper against the Nile tilapia genome sequence identified strong similarity to 169 tilapia LG-anchored sequences and to 11 sequences of unknown location (UNK). One hundred fifty-eight of the scaffolds (93.5%) showed colocalization in both genomes. The macrosynteny relationships between the two species are presented in Figure 4. Unique correspondence was obtained for 18 LGs of white grouper and Nile tilapia. The largest grouper LG (LG1) showed 16 common BLASTN hits on the largest tilapia LG1 but also five additional hits on tilapia LG12. Grouper LG2 also had nine and eight hits on two tilapia LGs (LGs 12 and 22). Grouper LG6 had two hits on the largest tilapia nonanchored fragment (UNK1). In the other two cases, multiple hits were obtained for tilapia LG7 on grouper LGs 12 and 18 and for tilapia LG14 on grouper LGs 5 and 23.

### Macrosynteny between the white and orange-spotted groupers

**Figure 5:**
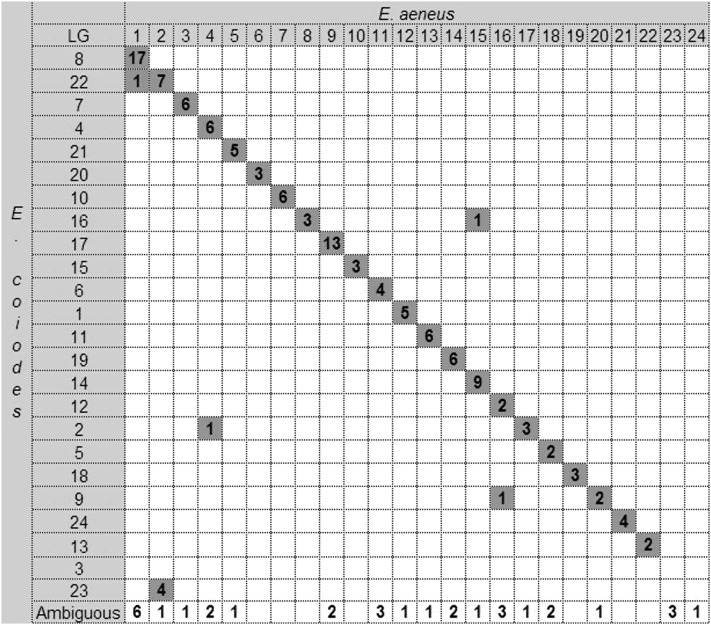
Macrosynteny relationships between linkage groups of white grouper and orange-spotted grouper. The numbers of white grouper scaffolds with significant BLASTN hits (E-value < 10^−10^) to sequences harboring SNP in orange-spotted grouper are presented. Putative syntenic pairs are indicated by gray boxes along the diagonal. The off-diagonal gray boxes represent synteny to additional LGs. A white grouper scaffold with multiple synteny relationships for different SNP sequences was assigned as “Ambiguous LG” at the bottom row.

Analysis of 5455 sequences of the orange-spotted grouper (84 bases stacks) by repeat maskers detected repetitive elements in 609 (11.2%) of them. BLASTN search of the remaining 4846 sequences against the *E. aeneus* assembled genomic database, detected 529 strong hits in 157 of the 177 largest scaffolds (E < 10^−10^). Analysis of colocalization of SNPs and microsatellites in the linkage maps of orange-spotted and white groupers identified corresponding LG pairs in the two genomes that are displayed on the diagonal of Figure 5. Correspondence was obtained for LGs 1−22 of the white grouper but not for LGs 23 and 24. Unique correspondence was obtained for 17 of the 22 LGs. For five white grouper LGs additional sporadic correspondence was found with additional orange-spotted LGs. However, only 47% of the SNP hits were in agreement with the previous correspondence. The similarity of 15 SNP sequences to additional eight scaffolds is displayed by the off diagonal squares in Figure 5, thus indicating mapping inconsistency between the genomes of both groupers. The remaining half of the SNP hits provided ambiguous information for the positions of 32 white grouper scaffolds (20.4%) assigned as “ambiguous LG” at the bottom row.

### Microsynteny between the white, kelp, and orange-spotted groupers

**Figure 6:**
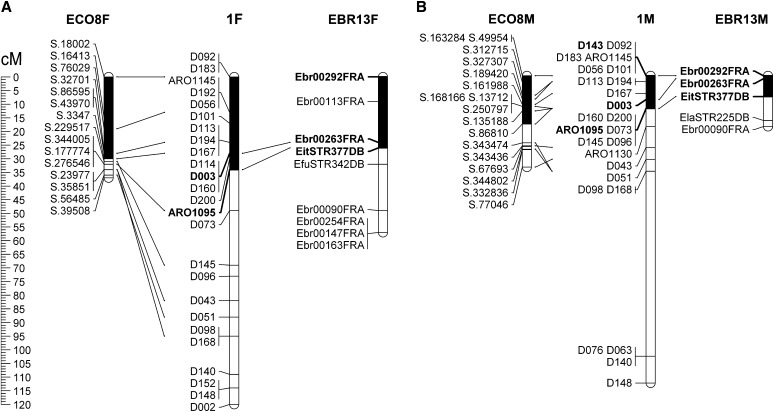
Comparative mapping of LG1 of the white grouper (center) and its syntenic LGs EBR13 of the kelp grouper (right), and ECO8 of the orange-spotted grouper (left) (adapted from Liu *et al.* 2013b and You *et al.* 2013). The three syntenic LGs in the different groupers are presented for female and male, in A and B, respectively. Three heterologous markers residing on both LG1 of the white grouper and EBR13 of the kelp grouper are presented in bold. All ECO8 single-nucleotide sequences that were located on scaffolds of the white grouper LG1 are presented. Marker names and positions (Kosambi centiMorgans) are indicated on the left of the LG delineations.

Analysis of 222 kelp grouper sequences (350−500 bases) by repeat masker detected repetitive elements in 72 (32.4%) of them. In contrast to the short sequences of orange-spotted grouper, long sequences of kelp grouper identified high similarity even after masking. Thus, BLASTN search identified strong similarity (E < 10^−10^) for 218 of 222 sequences in the white grouper assembled genomic database. Six of 146 (4.1%) sequences of the kelp grouper were similar to sequences in the white grouper large scaffolds. BLASTN search identified that 23 of 76 (30.3%) heterologous markers designed for kelp grouper originated from the same sequences that were used for the white grouper, and reported by us as ARO markers (Dor *et al.* 2014). Three of the 29 common markers of the white and kelp groupers were mapped to the corresponding LG1 and EBR13 (Figure 6). Seventeen markers of the white grouper and 34 linked SNP markers of the orange-spotted grouper supported the correspondence between LG1 and ECO8, respectively (Figure 6). Comparative mapping showed that the common markers were clustered at similar distances and with the same order in the corresponding LGs of the white, kelp, and orange-spotted groupers. By comparing the sex-specific maps, it is evident that the male maps were compressed relative to those of females in all of the three groupers (Figure 1, Figure 2, and Figure 6).

### Mapping position of TERRA-containing sequences

Eleven sequences contained both the (TTAGGG)_5_ and (GT)_10_ for development of markers that are localized to the chromosomal telomeres. Five of the 11 TERRA-containing markers were localized to ends of LGs, including markers D8881 and D9232, which mapped to two opposite ends of LG12 (Table 3). Mapping positions of the other TERRA-containing markers were ambiguous. Nevertheless, by comparing to random markers (Table 4), a significant enrichment of mapping TERRA-containing markers to LG ends was exemplified (*P* = 5 × 10^−39^).

**Table 3:** Mapping positions of TERRA-containing markers*^a^*

		Localization Within LG**^b^**	
Marker	LG	Female	Male
D002	1	E	ND
D9828	3	I	E
D10282	4	I	I
D046	7	nE	E
D211	9	nE	nE
D7527	11	I	I
D8881	12	E	ND
D9232	12	E	E
D360	14	nE	nE
D009	15	I	I
D606	20	nE	nE

a TERRA, telomeric DNA that are capable of encoding repeat-containing RNA (Lejnine *et al.* 1995).

b LG, linkage group; E, end; ND, not determined; nE, near end; I , internal.

**Table 4:** Enrichment of mapping TERRA-containing markers to linkage groups ends*^a^*

	Type of Markers		Total
Mapping position in LG	Random*^b^*	TERRA*^c^*	
End	43	5	48
Internal	149	6	155
Total	192	11	203

a χ^2^ probability of 5 × 10^−39^

b Selected at random in the genome.

c Telomeric DNA that are capable of encoding repeat-containing RNA (Lejnine *et al*. 1995).

## Discussion

In captive broodstock with multiple potential parents of the white grouper, an effective test for parenthood identification is required. In a previous study, we developed microsatellite markers that can be used for parenthood analysis (Dor *et al.* 2014). In this study we used 34 of these markers for parenthood identification of 12 potential parents and 80 presumed offspring obtained in two subsequent spawns. All exclusions of individuals were based on at least two markers and a maximum of 22 markers with conflicting genotypes between parents and putative progeny. The exclusion of parenthood by number of markers is presented in Figure S2. Eight of the 34 markers were highly informative by excluding 85% of individuals. Thus, only a few additional polymorphic markers are needed to establish a set of markers with high statistical power for parenthood exclusion (Jamieson and Taylor 1997). Among the 12 potential parents, two males and two females were identified as the actual parents. Moreover, we demonstrated a reproductive dominance of a single male in captivity through three subsequent spawning events. It is not known whether such reproductive behavior also applies in the wild similar to that of the red hind grouper (*E. guttatus*) (Shapiro *et al.* 1993), or to the single pair mating observed for a wild population of the Mediterranean dusky grouper (*E. marginatus*) (Zabala *et al.* 1997).

Comparison of the efficiency of development of heterologous and homologous microsatellite markers for the white grouper, a species without a sequenced genome, showed an overall higher efficiency for homologous (95%) over heterologous (63%) markers (Table 5). There was a greater efficiency of construction of heterologous markers originating from closely (83%) rather than distant (42%) related species (Dor *et al.* 2014).

**Table 5:** Comparison of rate of polymorphism between hetero- and homologous microsatellites markers in the white grouper

	No. Markers*^a^*		
Type of Markers	Tested	Polymorphic	Polymorphism Rate, %
Heterologous*^b^*	222	140	63.1
Homologous	198	188	94.9

a χ^2^ probability for difference in rate of polymorphic markers (*P* = 2 × 10^−63^).

b Dor *et al.* 2014.

The white grouper karyotype is composed of 24 pairs of telocentric chromosomes similar to other *Epinephelus* species (Gorshkova *et al.* 2002; Klinkhardt *et al.* 1995). In the current study the first-generation linkage map of the white grouper was constructed based on genotyping data of 222 microsatellites. The markers were assigned to 24 linkage groups. The female and male maps spanned 1053 and 886 cM, with an average intermarker distance of 5.8 and 5.0 cM, respectively. Therefore, the mapping density of this map would be sufficient for mapping of loci affecting economical traits. Interestingly, a similar map density was constructed for the salt tolerant tilapia and the kelp grouper, with mapping populations consisting of 95 and 90 individuals, respectively, compared with 48 in this study (Cnaani *et al.* 2003; Liu *et al.* 2013a). Apparently, the parents were heterozygous for most of the markers analyzed in the current study, thus providing information on both male and female recombination events for construction of linkage maps. Furthermore, a significant enrichment of localization of markers to LG ends was obtained by developing TERRA-containing markers. However, at least three such markers mapped to internal regions of LGs in accordance to the general phenomenon in vertebrates (Moyzis *et al.* 1988). A high-density genetic linkage map based on thousands of markers was recently constructed for the orange-spotted grouper (You *et al.* 2013). A cluster of 16 linked SNP markers that mapped to LG1 of the white grouper was demonstrated as a model for the potential of integration of markers across groupers (Figure 6).

Two markers and their adjacent neighbors on LGs 1 and 4 showed non-Mendelian cosegregation that may indicate the existence of deleterious effects. However, the partial lethal effects do not explain the extremely low larval viability of <0.1% inspected in the white grouper hatchery. Interestingly, the dense map of the orange-spotted grouper harbors at least eight distinct and large regions with absence of localized markers (You *et al.* 2013). Removal from the map of SNP markers that deviated from expected Mendelian segregation may have caused these “marker deserts” signifying potential regions with deleterious effects.

We found a female/male recombination ratio of 1.19:1 which is different from the 1.45:1 and 1.03:1 ratios reported for the kelp and orange-spotted groupers, respectively (Liu *et al.* 2013b; You *et al.* 2013). In the white grouper the female map is significantly longer for 13 LGs, whereas the male map is longer for 6 other LGs. In mammals, it was shown that differences in recombination rate appear in specific chromosomal segments (Weitkamp *et al.* 1971). Gender-specific rate of meiotic recombination is prevalent in animal species, but the underlying mechanisms remain unknown. Haldane’s general rule assumes that the heterogametic sex, which is male in mammals, is characterized with reduced recombination (Haldane 1922). In most fish species no heteromorphic chromosomes were detected although male recombination is reduced. Nevertheless, the genetic mechanism of sex determination is controlled by the XX/XY or WZ/ZZ systems. For halibut and grass carp with the XX/XY system, the female map was longer than that in males, while in half-smooth tongue sole with the WZ/ZZ system, the male map was longer (Reid *et al.* 2007; Xia *et al.* 2010; Song *et al.* 2012). Thus, the compression in female or male recombination rate is more likely associated with the W or Y heterogametic region, respectively, rather than the whole sex chromosome morphology. This conclusion is supported by the detection of genes that regulate the rate of crossing-over on the Y chromosome of *Drosophilla apapassea* (Moriwaki *et al.* 1979). However, the type of recombination rate depends on the actual gender of the individual as implicated from the experiments of Yamamoto (1961) who observed a fivefold recombination increase after sex reversal of medaka male (*Oryzias latipes*).

All individuals of hermaphroditic protandrous and protogyneous species develop primarily into males and females, respectively, possibly due to absence of genetic sex determination. However, Franch *et al.* (2006) demonstrated that in protandrous gilthead sea bream the female:male recombination ratio is 1.2:1, similar to that found in the current study for the white grouper. Moreover, they identified QTL for sex determination in this hermaphrodite species. Thus, it may be speculated that protandrous and protogyneous species also have cryptic genetic sex-determination systems that are realized at a later stage of development, as the potential for sex reversal from the predetermined gender. QTL for sex determination were found in two tilapia species *e.g.*, *O. niloticus*, *O. aureus*, and their hybrids on LGs 1, 3, and 23 (Shirak *et al.* 2002; 2006; Lee *et al.* 2003; 2004; Eshel *et al.* 2011; 2012), which relate to those in the white grouper on LGs 1, 19, and 21, respectively. Macrosynteny relationships between the white grouper and 24 tilapia LGs, that represent 22 subtelocentric chromosomes (Ocalewicz *et al.* 2009), showed unequivocal colocalization for most LGs (18/24), in accordance with the high synteny observed between orange-spotted grouper and tilapia (You *et al.* 2013). Thus, additional heterologous markers already mapped on LG23 of tilapia may be tested for localization on LG21 of the white grouper, for mapping sex-determining loci.

Macrosynteny relationships between the white grouper and orange-spotted grouper LGs showed unequivocal co-localization for most LGs (22/24). Furthermore, three linked markers on LG1 of the white grouper showed similar order and inter-marker recombination distances in the kelp grouper (Liu *et al.* 2013b). Likewise, 15 SNP markers of the orange-spotted grouper (You *et al.* 2013) were assigned to a cluster of markers on LG1 of the white grouper. Thus, due to high synteny among groupers, it would be useful to integrate the markers that were developed for different groupers, depending on sharing of sequence data, into a comprehensive consensus map.

## Supplementary Material

Supporting Information
