# Supplementary material for: Construction of a Microsatellites-Based Linkage Map for the White Grouper (Epinephelus aeneus)
Source: G3 (Bethesda). 2014 Jun 5;4(8):1455–64. doi: 10.1534/g3.114.011387 (PMC4132176; doi:10.1534/g3.114.011387)
Supplement: Supporting Information [file supp_4_8_1455__index.html]

Construction of a Microsatellites-Based Linkage Map for the White Grouper (Epinephelus aeneus) — Supporting Information 

# Construction of a Microsatellites-Based Linkage Map for the White Grouper (*Epinephelus aeneus*)

## Supporting Information for Dor *et al.*, 2014

**Files in this Data Supplement:**

- Supporting Information - Figures S1-S2 and Tables S1-S3 (PDF, 245 KB)
- Figure S1 - Genotyping by fragment analysis is illustrated for D078 microsatellite marker; heterozygous sire (188/196) and dam (188/192), and the four possible genetic combinations resulting in their progeny. (PDF, 404 KB)
- Figure S2 - Exclusion of parenthood by number of markers. (PDF, 331 KB)
- Table S2 - Number of offspring of two males and two females in two subsequent spawns as verified by parenthood identification using 34 microsatellite markers. (PDF, 108 KB)
- Table S3 - Origin of microsatellite markers used for linkage map construction. (PDF, 108 KB)
- Table S1 - Markers used for construction of tilapia linkage maps. (.xls, 241 KB)
