# Supplementary material for: Construction of a Microsatellites-Based Linkage Map for the White Grouper (Epinephelus aeneus)
Source: G3 (Bethesda). 2014 Jun 5;4(8):1455–64. doi: 10.1534/g3.114.011387 (PMC4132176; doi:10.1534/g3.114.011387)
Supplement: Supporting Information [file supp_g3.114.011387_011387SI.pdf]

G3 · Genes | Genomes | Genetics

Construction of a Microsatellites-Based Linkage Map for the White Grouper (*Epinephelus aeneus*)

Lior Dor[*†], Andrey Shirak[*], Sergei Gorshkov[‡], Mark R. Band[§], Abraham Korol[**], Yefim Ronin[**], Arie Curzon[*], Gideon Hulata[*], Eyal Seroussi[*] and Micha Ron[*1]

[*]Institute of Animal Science, Agricultural Research Organization, Bet Dagan 50250, Israel
[†]Robert H. Smith Faculty of Agriculture, Food and Environment, Hebrew University of Jerusalem, Rehovot 76100, Israel
[‡]National Center for Mariculture, Israel Oceanographic and Limnological Research, Eilat 88112, Israel
[§]The Carver Biotechnology Center, University of Illinois, Urbana, IL 61801, USA
[**]University Haifa, Faculty of Science, Institute of Evolution, Haifa 31905, Israel
[1]Corresponding author

Raw sequencing data of White grouper from this article was deposited under GenBank accession number PRJEB5936

Corresponding author details: Micha Ron; Institute of Animal Science, Agricultural Research Organization, Bet Dagan 50250, Israel; +97289484442 micha.ron@mail.huji.ac.il

DOI: 10.1534/g3.114.011387

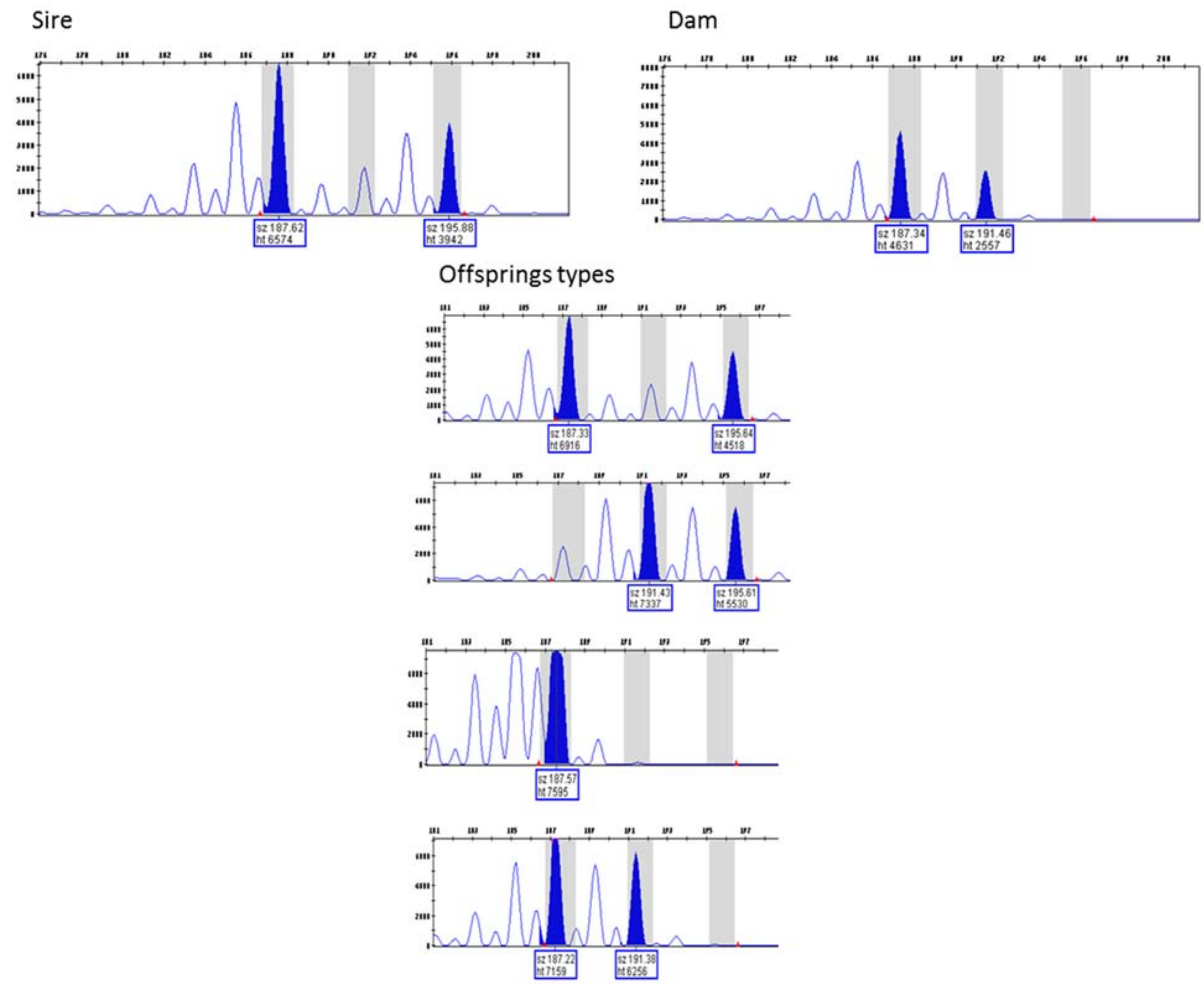

Figure S1 Genotyping by fragment analysis is illustrated for D078 microsatellite marker; heterozygous sire (188/196) and dam (188/192), and the four possible genetic combinations resulting in their progeny. Automatic genotyping is based on fixed positions of three bins (grey fields) representing the three alleles.

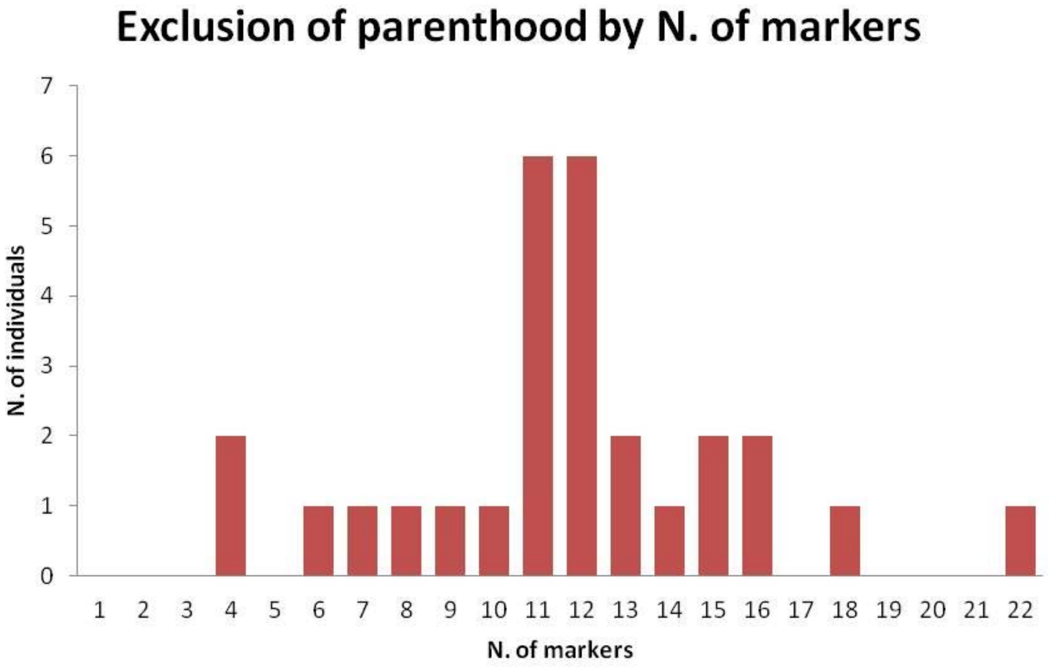

Figure S2 Exclusion of parenthood by number of markers.

Table S1 Markers used for construction of tilapia linkage maps.

Available for download as an Excel file at http://www.g3journal.org/lookup/suppl/doi:10.1534/g3.114.011387/-/DC1

Table S2 Number of offspring of two males and two females in two subsequent spawns as verified by parenthood identification using 34 microsatellite markers.

Male		M2		M4	
Spawn		1st	2nd	1st	2nd
Female	F9	37	15	5	0
	F11	6	14	0	3

Table S3 Origin of microsatellite markers used for linkage map construction.

Origin of microsatellite markers		No.
Heterologous[1]		40
Next generation sequencing	Largest scaffolds	177
	TERRA containing scaffolds[2]	11
Total		**228**

[1]Dor et al. (2014)

[2]Telomeric repeat-containing RNA (TERRA) (Lejnine et al. 1995).
