# Supplementary material for: Construction of a Microsatellites-Based Linkage Map for the White Grouper (Epinephelus aeneus)
Source: G3 (Bethesda). 2014 Jun 5;4(8):1455–64. doi: 10.1534/g3.114.011387 (PMC4132176; doi:10.1534/g3.114.011387)
Supplement: Supporting Information [file supp_g3.114.011387_FigureS1.pdf]

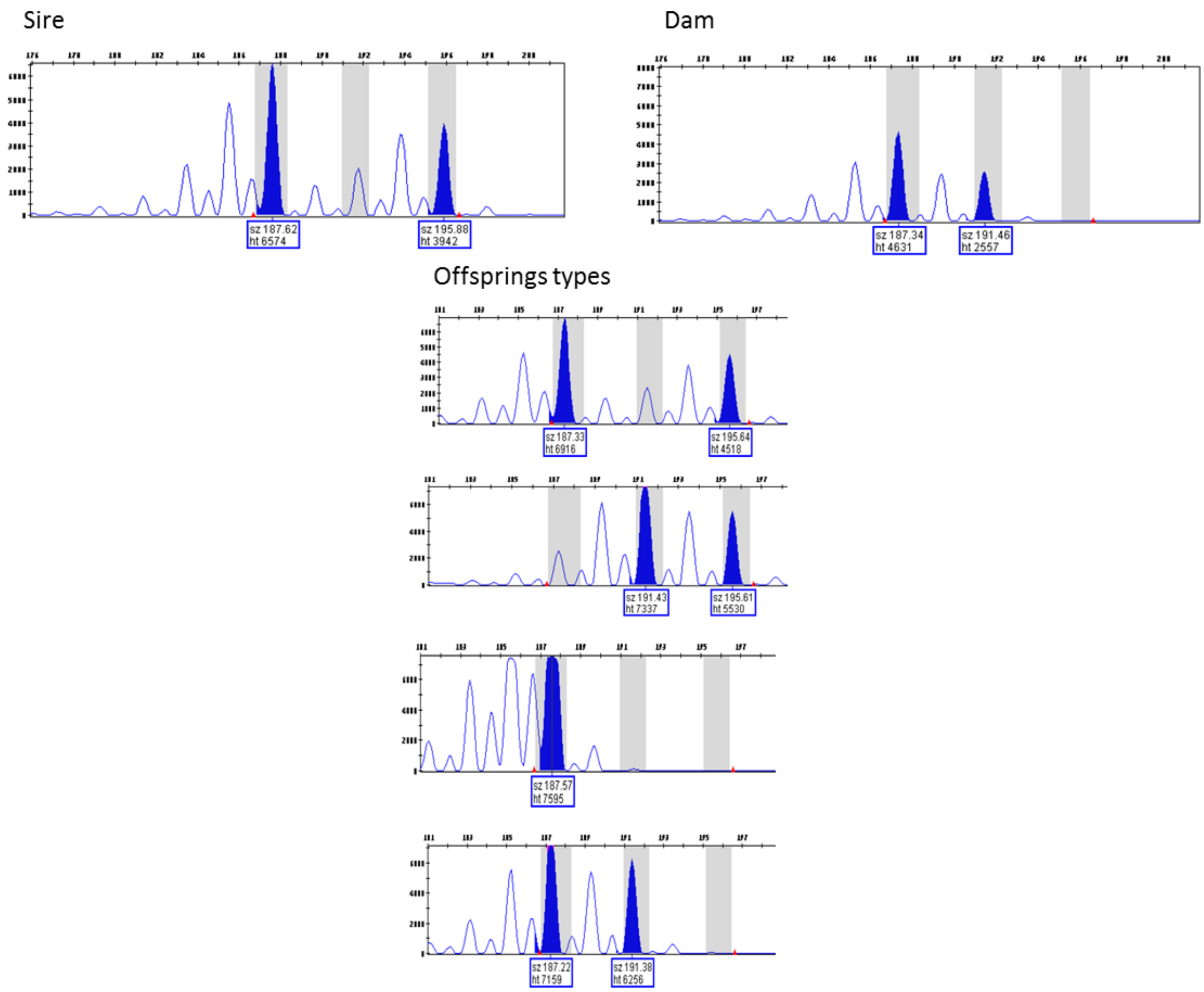

Figure S1 Genotyping by fragment analysis is illustrated for D078 microsatellite marker; heterozygous sire (188/196) and dam (188/192), and the four possible genetic combinations resulting in their progeny. Automatic genotyping is based on fixed positions of three bins (grey fields) representing the three alleles.
