# Supplementary figures and images for: Construction of a Microsatellites-Based Linkage Map for the White Grouper (Epinephelus aeneus)
Source: G3 (Bethesda). 2014 Jun 5;4(8):1455–64. doi: 10.1534/g3.114.011387 (PMC4132176; doi:10.1534/g3.114.011387)

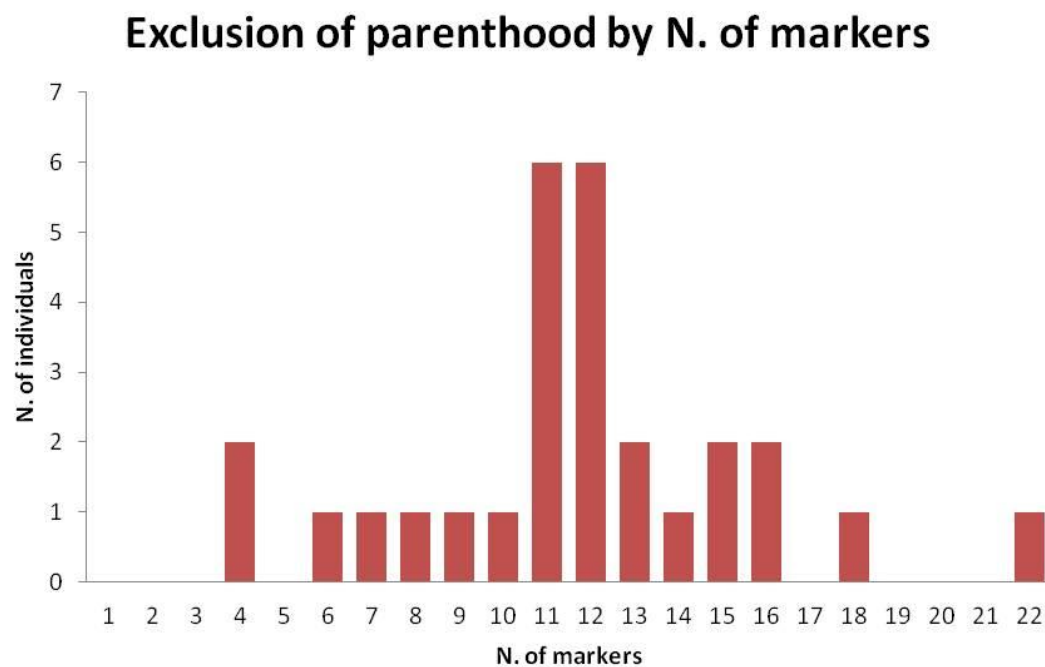

**Figure S2** Exclusion of parenthood by number of markers.

Supplement: Supporting Information [file supp_g3.114.011387_FigureS2.pdf]
