# Supplementary material for: Construction of a Microsatellites-Based Linkage Map for the White Grouper (Epinephelus aeneus)
Source: G3 (Bethesda). 2014 Jun 5;4(8):1455–64. doi: 10.1534/g3.114.011387 (PMC4132176; doi:10.1534/g3.114.011387)
Supplement: Supporting Information [file supp_g3.114.011387_TableS2.pdf]

Table S2 Number of offspring of two males and two females in two subsequent spawns as verified by parenthood identification using 34 microsatellite markers.

Male		M2		M4	
Spawn		1st	2nd	1st	2nd
Female	F9	37	15	5	0
	F11	6	14	0	3
