# Supplementary material for: Construction of a Microsatellites-Based Linkage Map for the White Grouper (Epinephelus aeneus)
Source: G3 (Bethesda). 2014 Jun 5;4(8):1455–64. doi: 10.1534/g3.114.011387 (PMC4132176; doi:10.1534/g3.114.011387)
Supplement: Supporting Information [file supp_g3.114.011387_TableS3.pdf]

Table S3 Origin of microsatellite markers used for linkage map construction.

Origin of microsatellite markers		No.
Heterologous[1]		40
Next generation sequencing	Largest scaffolds	177
	TERRA containing scaffolds[2]	11
Total		**228**

[1]Dor et al. (2014)

[2]Telomeric repeat-containing RNA (TERRA) (Lejnine et al. 1995).
